# MicroRNA-17-92a-1 Host Gene (MIR17HG) Expression Signature and rs4284505 Variant Association with Alopecia Areata: A Case–Control Study

**DOI:** 10.3390/genes13030505

**Published:** 2022-03-12

**Authors:** Salwa Faisal, Eman A. Toraih, Lina M. Atef, Ranya Hassan, Marwa M. Fouad, Essam Al Ageeli, Manal S. Fawzy, Hussein Abdelaziz Abdalla

**Affiliations:** 1 Department of Medical Biochemistry and Molecular Biology, Faculty of Medicine, Suez Canal University, Ismailia 41522, Egypt; dr_salwafaisal@yahoo.com; 2 Division of Endocrine and Oncologic Surgery, Department of Surgery, School of Medicine, Tulane University, New Orleans, LA 70112, USA; 3 Genetics Unit, Department of Histology and Cell Biology, Faculty of Medicine, Suez Canal University, Ismailia 41522, Egypt; 4 Department of Dermatology, Venerology and Andrology, Faculty of Medicine, Suez Canal University, Ismailia 41522, Egypt; Lina_atef@yahoo.com; 5 Department of Clinical Pathology, Faculty of Medicine, Suez Canal University, Ismailia 41522, Egypt; rania.moustafa@med.suez.edu.eg; 6 Department of Microbiology and Immunology, Faculty of Medicine, Suez Canal University, Ismailia 41522, Egypt; marwa_fouad80@med.suez.edu.eg; 7 Department of Clinical Biochemistry (Medical Genetics), Faculty of Medicine, Jazan University, Jazan 82911, Saudi Arabia; dr.ageeli@gmail.com; 8 Department of Biochemistry, Faculty of Medicine, Northern Border University, Arar 91431, Saudi Arabia; 9 Department of Medical Biochemistry, Faculty of Medicine, Taibah University, Al Madinah Al Munawarah 41311, Saudi Arabia; huss166@hotmail.com; 10 Department of Medical Biochemistry, Faculty of Medicine, Mansoura University, Mansoura 35516, Egypt

**Keywords:** alopecia areata, gene expression, gene polymorphism, MIR17HG, Real-Time PCR

## Abstract

Accumulating evidence indicates the implication of microRNAs (miRs) in cutaneous and hair follicle immunobiology. We evaluated, for the first time, the miR-17-92a-1 cluster host gene (MIR17HG) expression in peripheral blood of 248 unrelated alopecia areata (AA) patients compared to 244 matched controls using Real-Time qPCR. We also tested its association with different rs4284505A>G genotypes (based on TaqMan allelic discrimination PCR) and the available clinical data. The adjusted odds ratio (OR) and 95% confidence interval (CI) were calculated for each genetic association model. The upregulation of miR-17 was observed in the serum of patients with alopecia compared to controls (*p*-value = 0.004). The ROC curve showed high diagnostic performance of miR-17 in differentiating between patients and controls (AUC = 0.85, *p*-value < 0.001). rs4284505*A/G heterozygotes were more susceptible to the disease (OR = 1.57, 95% CI = 1.01–2.45) under the over-dominant model. Interestingly, patients with the rs4284505*G/G genotype had a higher level of miR-17 than those with the A/A and A/G genotypes. The G/G genotype was associated with the severe phenotype (*p*-value = 0.038). A/G carriers were the youngest (*p*-value < 0.001), had more frequent scalp infection (*p*-value = 0.006), exhibited the worst dermatology life quality index score (*p*-value = 0.037), and responded less to treatment (*p*-value = 0.033). In conclusion, MIR17HG expression and the rs4284505 variant were significantly associated with AA and could play a role in pathogenesis and phenotype in the Egyptian population. Further multi-center studies in other ethnicities are warranted to replicate the findings.

## 1. Introduction

Alopecia areata (AA) is an autoimmune, non-scarring hair loss with the preservation of the hair follicle. It has a heterogeneous clinical pattern that can take many forms, from well-defined bald patchy lesions (patchy AA) to total hair loss on the scalp (alopecia totalis) or whole body (alopecia universalis) [1]. The estimated prevalence of AA is 1 in 1000 people, with nearly 2% lifetime risk [2]. Numerous mechanisms have been proposed, and multiple risk factors have been proposed, such as emotional stress, trauma, thyroid disease, and hormonal fluctuations [1]. Skin biopsies of affected lesions showed lymphocytic infiltration around the hair bulb [3]. Genome-wide association studies in animal models and patients depicted AA as a complex polygenic disorder with several genetically susceptible loci enclosing high-risk candidate genes [1,4,5]. Alopecia areata is challenging to manage medically; therefore, a new targeted therapy is warranted to overcome the hurdles encountered during management.

With the new era of non-coding RNAs, which virtually fine-tune all cellular biological processes and signaling pathways, a new prospective field has risen on the scene [6]. This type of non-coding ribonucleic acid molecule can be stratified according to the nucleotides (nts) size into short RNAs (<200 nts) and long non-coding RNAs (lncRNAs; >200 nts) [7]. The short non-coding sub-type microRNAs (miRNAs) are implicated in the temporal- and spatial-specific regulation of all body organs and tissues, including skin morphogenesis and hair follicle cycling and growth [8,9]. Recent evidence highlighted the importance of exploring this type of non-coding RNA to unravel the underlying pathophysiological mechanisms of AA and to discover novel biomarkers that identify its severity, differentiate its subtypes, or predict the therapeutic response [10,11,12,13].

**Figure 1:**
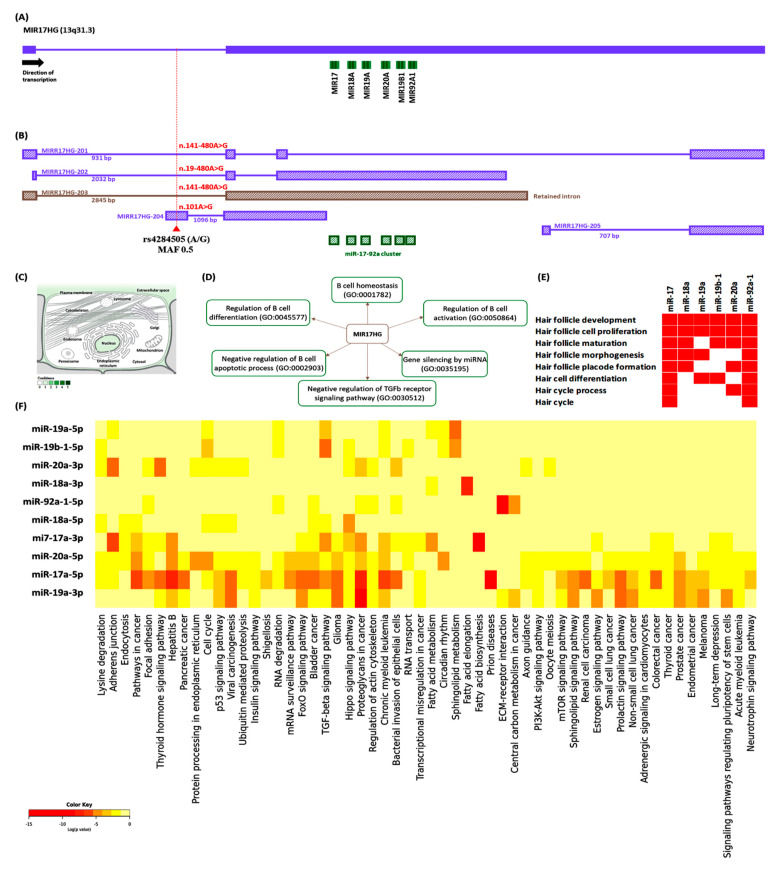
Structural and functional analysis of the MIR17HG cluster region. (**A**) Genomic context of the MIR17 host gene. The MIR17HG cluster gene is located at chromosome 13q31.3 at the forward strand. The genomic sequence enclosed six microRNAs with the following order: miR-17, 18A, 19A, 20A, 19B1, and 92A1. (**B**) MIR17HG transcripts and polymorphism. The gene encodes four lncRNAs and one retained intron transcript by alternative splicing, but only the longest (MIR17HG-202) is a polycistronic transcript containing the miR-17-92a-1 cluster. A common variant, rs4284505, caused by A with G substitution with a minor allele frequency of 0.5 was analyzed in the study. It was overlapped with four MIR17HG transcripts: within the first intron of three of them (480 bases upstream to the start of the second exon) and as a non-coding exon SNP in another one at nucleotide number 101 out of 1096 of the first exon. (**C**) Subcellular localization of MIR17HG within the nucleus and extracellular matrix. The color code indicates the confidence level of abundance (**D**) gene ontology analysis of MIR17HG. (**E**) Six microRNA cluster is involved in hair follicle-related biological processes. (**F**) Functional enrichment KEGG pathway analysis for the six microRNAs within the MIR17HG cluster. [Data source: (www.ensembl.org), (http://diana.imis.athena-innovation.gr/), and (www.GeneCards.org) (all web sites are last accessed on 25 August 2021)].

About 10% of miRNA genes are located within the introns and exons of lncRNAs. The gene that contains the miRNA coding sequence is called the miRNA host gene (HG) [14]. A group of six miRNAs (miR-17, miR-18a, miR-19a, miR-20a, miR-19b-1, and miR-92a-1) is annotated at chromosome 13q31.3 within the genomic context of the miR-17-92a-1 Cluster Host gene (MIR17HG). This gene encodes four lncRNAs and one retained intron transcript by alternative splicing (www.ensembl.org, accessed on 25 August 2021), but only the longest is a polycistronic transcript containing the miR-17-92a-1 cluster (Figure 1). The MIR17HG gene is involved in cell growth and differentiation [15]. Gene ontology analysis depicted the MIR17HG gene as a key player in B cell hemostasis, activation, and differentiation (www.Genecards.org accessed on 25 August 2021). Additionally, microRNAs originating from the post-transcriptional splicing of the MIR17HG were enriched in hair follicle-related pathways (Table S1). Functional enrichment pathway analysis of the six-miRNA cluster revealed them to be involved in the 56 Kyoto Encyclopedia of Genes and Genomes (KEGG) signaling pathways and 143 biological processes, and some involved in the inflammatory response and cell-cell signaling (http://diana.imis.athena-innovation.gr/ accessed on 25 August 2021) (Table S2).

Gene polymorphism refers to different genomic sequences at a specific location in the population. A common variant (rs4284505) exists within the MIR17HG gene with a minor allele frequency (MAF) of 0.5. It was previously cited to be in linkage disequilibrium with an upstream polymorphism related to multiple sclerosis [16] and to be associated with systemic lupus erythematosus (SLE) [17]. The impact of this polymorphism on AA susceptibility and phenotype has not been studied before, especially in the present population. In this sense, the authors were inspired to investigate the association of rs4284505 polymorphism in the MIR17HG gene cluster with AA risk and severity. The circulatory miR-17 expression level was also profiled for genotype–phenotype correlation to test its putative role as a diagnostic or prognostic molecular biomarker for this disease.

## 2. Materials and Methods

### 2.1. Study Participants

Four hundred and ninety-two unrelated subjects were included in this case–control study (248 alopecia areata patients and 244 matched healthy controls). The patients were recruited from the Dermatology Outpatient Clinic from four cities (Port-said, Suez, Ismailia, and Cairo) in Egypt between December 2017 and March 2019. Patients who use topical treatment were not excluded as long as there was no significant hair regrowth. The exclusion criteria included cohorts receiving systemic treatment within less than two weeks of sampling. Additionally, patients known to have tumors, whether malignant or benign, or viral infection, such as HCV, HBV, or HIV, were excluded.

The control group was recruited from healthy blood donors attending the blood bank of the specified hospitals (i.e., the same hospital/clinic distribution from which the patients were recruited). The exclusion criteria included being a relative of the individuals in the alopecia areata patients or having a history of other autoimmune diseases, such as inflammatory bowel disease, thyroid disease, systemic lupus erythematosus, psoriasis, or vitiligo. Upon taking their consent to participate as a control subject in the study, they underwent thorough history taking and physical examination to exclude incidental alopecia lesions.

The study was conducted following the guidelines in the Declaration of Helsinki and had the approval of the Ethics Committee of the Faculty of Medicine, Suez Canal University. Informed consent was obtained from all participants.

### 2.2. Clinical Assessment

The study participants underwent complete history taking, and general and dermatological examination. Detailed histories of the family and comorbid autoimmune diseases were obtained. Patients with age at diagnosis of ≤20 years were considered as early-onset disease, while disease duration spanning more than 12 months was categorized as having prolonged duration. AA patients were assessed for severity via the Severity of Alopecia Tool II (SALT) score, devised by the National Alopecia Areata Foundation “NAAF” working committee [18], as described in our previous publication [19]. Poor prognostic factors, such as young age at onset, family history of alopecia, atopy, severe phenotype, such as ophiasis or alopecia totalis and universalis (AT/AU), prolonged duration, nail disease, or associated autoimmune disease were scored, and the sum was used for comparison between patients [20].

### 2.3. Allelic Discrimination of rs4284505 Variant

Seven milliliters of peripheral blood were collected under aseptic conditions. Five milliliters were taken using EDITA anticoagulant vacutainer tubes for genomic DNA extraction from the buffy coat after sample centrifugation using a QIAamp DNA Blood Mini kit (Catalog no. 51104; Qiagen, Hilden, Germany) following the manufacturer’s instructions. The other two milliliters were collected in serum-separating vacutainers to obtain the serum for subsequent gene expression profiling.

The extracted DNA concentration and purity were evaluated using “NanoDrop ND-1000 (NanoDrop Technologies, Inc. Wilmington, DE, USA)”. The MIR17HG rs4284505 polymorphism was genotyped by the TaqMan genotyping polymerase chain reaction (PCR) using StepOne Real-Time (Applied Biosystems, Thermo Fisher Scientific, Foster City, CA, USA). The applied protocol was run blindly to the case/control status with a final volume of 20 μL containing the extracted genomic DNA (20 ng), TaqMan SNP Genotyping Assay Mix (1 μL) (assays ID: C__26557482_10, Applied Biosystems), and TaqMan Universal PCR Master Mix (10 μL) (Catalog no. 4371353) [17]. Two-allele-specific TaqMan minor groove-binding (MGB) probes containing distinct fluorescent dyes were included in the genotyping assay. The context sequence of interest for the specified variant was [VIC/FAM]: CTGTTCCTAAACTGCACAAAGGGA[A/G]AAGGAACTGAAAAAGGCA GGCTCGT. The following thermocycler program was followed: 95 °C for denaturation (10 min), followed by 40 cycles at 90 °C (15 s) and 60 °C (1 min). Proper non-template and no-enzyme negative controls were applied in each run to ensure the absence of contamination (Figure S1A).

The authors performed an initial run to select samples with different genotypes, including two homozygous for wild/mutant genotypes and one heterozygous genotype. These three genotypes were run throughout the experiment as controls for every run. The pre-designed TaqMan assay applied in the present work included sequence-specific forward and reverse primers to amplify the polymorphic sequence of interest and, together with two TaqMan minor groove binder (MGB) probes with nonfluorescent quenchers, ensured the success of the reaction. Therefore, the possibility of false-positive results was decreased [21]. Ten percent of the included samples were randomly allocated for assay replication with a 100% concordance rate.

### 2.4. MIR17HG Expression Profiling

The expression level of MIR17HG (i.e., has-miR-17-5p) was determined in a subset of our total study population (144 alopecia cases and 86 controls). The total RNA, including small RNA, was extracted from the serum samples using a Qiagen miRNeasy Serum/Plasma Kit (Catalog no. 217184, Qiagen) as described by the manufacturer. After the assessment of RNA quality and purity (as described above), reverse transcription was specifically generated using 5x of microRNA-specific stem-loop reverse-transcriptase (RT) primer (assay ID 002308, Applied Biosystems) and the RT (P/N 4366596, Applied Biosystems) [22,23]. A small nuclear RNA U6 (RNU6B) primer set (Applied Biosystem, assay ID: 001093, cat. no 4427975) was used as an endogenous control. The RT reactions were performed in a Mastercycler Gradient Thermocycler (Eppendorf, Hamburg, Germany) to amplify the cDNA. Then, MIR17HG was quantified by the Real-Time PCR protocol using an AB 7500HT instrument with SDS Software version 2.1.1 (Applied Biosystems) (Figure S1B), using the TaqMan MicroRNA assay (20×) (Applied Biosystems, assay ID 002308) and TaqMan Universal PCR Master Mix II, No Uracil-N glycosylase (UNG) (2×) (P/N 4440043, Applied Biosystems) in a final volume of 20 µL, including “1.33 µL RT products, 2 × TaqMan Universal Master Mix II, 1 µL TaqMan MicroRNA assay/RNU6B assay”. The PCR program was 95 °C (10 min), followed by 40 cycles of 92 °C (15 s) and 60 °C (1 min). No-template (NT) samples were included as negative controls in each run. The minimum information for the publication of quantitative real-time PCR experiments (MIQE) guidelines was followed [24].

### 2.5. Statistical Analysis

Statistical analysis was performed using SPSS version 26.0 (IBM Corp., Armonk, NY, USA). The LIVAK method was used to calculate the fold change in MIR17HG after normalization to RNU6B. In brief, the quantification (threshold) cycle (C_q or_ C_T_) value was calculated as follows: gene relative expression = 2^−ΔΔCq^, where ΔΔC_q_ = (C_q_ MIR17HG − C_q_ RNU6B)_AA patients_ − (C_q_ MIR17HG − C_q_ RNU6B)_controls_ [25].

The allele and genotype frequencies were compared between the patients and controls using the Chi-square test as previously described [26]. The Hardy–Weinberg equilibrium (HWE) was estimated online (http://www.oege.org/software/hwe-mr-calc.shtml accessed on 25 August 2021). SNP analysis was conducted using the SNPstats web tool (www.snpstats.net accessed on 25 August 2021). Odds ratios (OR) with a 95% confidence interval (CI) were calculated for each genetic association model. Statistical differences between groups regarding their clinical features were tested using Chi-square, Fisher’s Exact, Mann–Whitney U, and Kruskal–Wallis tests. The Kolmogorov–Smirnov test was applied to test the normality of quantitative data. Receiver operator characteristics (ROC) analysis was run to evaluate the diagnostic performance of serum MIR17HG in AA patients. Multivariable analysis was conducted using binary logistic regression (enter method). Significantly correlated variables were removed after testing by Pearson’s correlation test. The Hosmer/Lemeshow test was used for the goodness-of-fit model. A two-tailed *p*-value of 0.05 was considered statistically significant.

## 3. Results

### 3.1. Characteristics of the Study Population

A total of 248 patients and 244 controls were enrolled in the study. Their mean age was 30.7 ± 7.1 years for the patients and 29.3 ± 5.4 for the controls. Most of the studied population were males, while females represented less than one-fifth. The baseline characteristics of the study population are provided in Table 1. There was no significant difference between the patients and controls regarding their demographic features and residency site.

**Figure 2:**
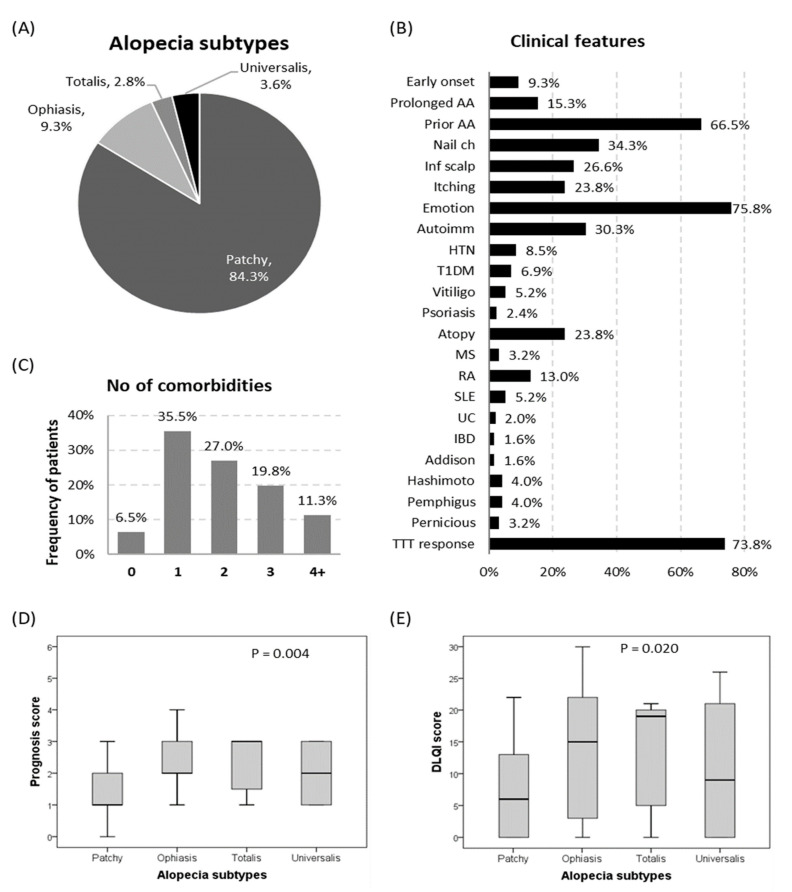
Disease characteristics of the alopecia cohorts. (**A**) Alopecia subtypes. (**B**) Clinical features of patients with AA. (**C**) Frequency of AA patients with different disease comorbidities. (**D**) Prognosis score among different alopecia clinical subtypes. (**E**) Dermatology Life Quality Index score among different alopecia clinical subtypes. The Kruskal–Wallis test was used. *p*-values < 0.05 were considered statistically significant. AA: alopecia areata or other autoimmune disorders; nail ch: nail changes; Inf scalp: scalp infection; HTN: hypertension; T1DM: type 1 diabetes mellitus; MS: multiple sclerosis; RA: rheumatoid arthritis; SLE: systemic lupus erythematosus; UC: ulcerative colitis; IBD: inflammatory bowel disease; emotion: emotional or psychological problem; TTT: treatment; and DLQI: Dermatology Life Quality Index.

Approximately 84% of alopecia patients had patchy scalp lesions of sudden onset and progressive course; 23 patients (9.3%) exhibited the ophiasis pattern, while seven (2.8%) and nine (3.6%) of the patients exhibited alopecia totalis and universalis, respectively. As depicted in Figure 2, two-thirds had a prior episode of alopecia, and nearly one-third had associated nail changes. Almost all patients had concomitant comorbidity disease, particularly insulin-dependent type 1 diabetes, vitiligo, rheumatoid arthritis, and SLE. Patients with the severe phenotype (alopecia totalis and alopecia universalis) had worse quality of life scores, higher SALT scores, and a poor prognostic index (Table 2).

### 3.2. Allelic Discrimination Analysis of rs4284505 SNP

The genotype frequencies of rs4284505 polymorphism in the patients and controls agreed with HWE (*p* > 0.05). MAF (A allele) accounted for 0.4. Comparing the patients and controls, both homozygote genotypes (A/A and G/G) were less frequent in the alopecia patients (Table 3). The genetic association models showed that A/A conferred protection against developing alopecia (OR = 0.51, 95% CI = 0.27–0.96) under the recessive model, while heterozygote genotype (A/G) carriers were more susceptible to the disease (OR = 1.57, 95% CI = 1.01–2.45) under the overdominant model (Table 4).

### 3.3. Relative Expression of MIR17HG

**Figure 3:**
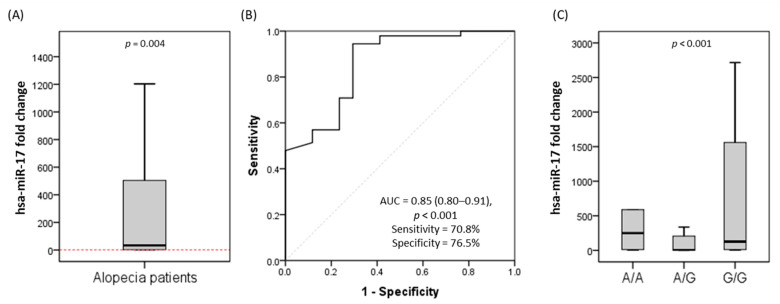
The relative expression levels of serum miR-17 in patients and controls. (**A**) Fold change in the alopecia patients compared to controls. Values are represented as the medians. The box defines the upper and lower quartiles (25% and 75%, respectively), and the error bars indicate the upper and lower adjacent limits. The red dotted line indicated the control group’s expression level. The Mann–Whitney U test was used. (**B**) Receiver Operator Characteristics curve for differentiating between patients and the control. The model was significant (*p* < 0.001). (**C**) Comparing patients’ expression levels with different genotypes. The Kruskal–Wallis test was used. Pairwise comparisons were adjusted by the Bonferroni correction for multiple testing.

Over-expression of the levels of serum miR-17 was observed in alopecia patients compared to controls (*p* = 0.004). The ROC curve showed high diagnostic performance of miR-17 in differentiating between patients and controls (AUC = 0.85, *p* < 0.001) with high sensitivity and specificity (Figure 3). Patients with the rs4284505*G/G genotype had a higher level of miR-17 than those with the A/G genotype (*p* = 0.001). Similarly, miR-17 was significantly overexpressed in A/A carriers compared to heterozygotic patients (*p* = 0.014).

### 3.4. Association with Disease Characteristics

**Figure 4:**
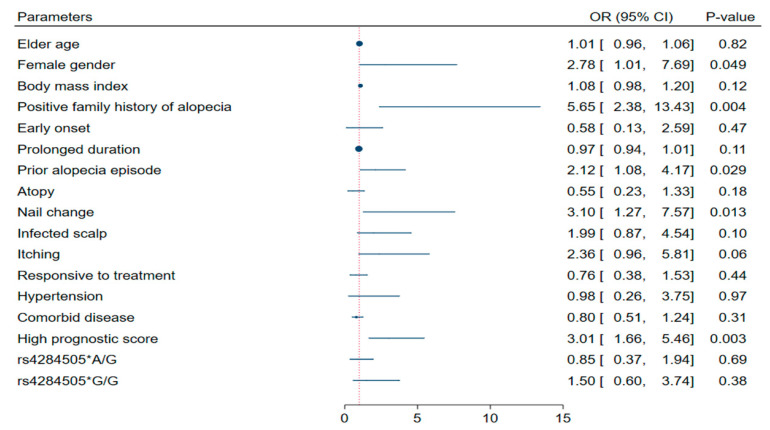
Independent predictors of severe alopecia disease. Multivariate analysis was conducted using binary logistic regression (enter method). Significantly correlated variables with each other were removed after testing by Pearson’s correlation test. The model correctly classified 64.5% of the observations; the Hosmer and Lemeshow test for goodness-of-fit was 0.203, indicating that the model was good. OR: odds ratio, CI: confidence interval, BMI: body mass index, FH: family history, TTT: treatment, and AA: alopecia areata.

Heterozygote patients (A/G) were the youngest (*p* < 0.001), had more frequent scalp infection (*p* = 0.006), exhibited the worst DLQI score (*p* = 0.037), and responded less to treatment (*p* = 0.033). In contrast, the G/G genotype was associated with a more severe phenotype (*p* = 0.038). Patients with concomitant diseases experienced higher levels of miR-17 than their counterparts (*p* = 0.049) (Table S3). Multivariable analysis revealed that female gender, positive family history of alopecia, associated nail changes, prior alopecia episodes, and high prognostic index were independent predictors of severe alopecia disease (Figure 4).

## 4. Discussion

Alopecia areata is a common hair loss disorder characterized by diversity in the disease phenotype and outcome. The development of AA involves a breakdown in hair follicle immune privilege followed by an autoimmune reaction. However, little is known about the underlying regulatory molecular mechanisms that could pave the way for proper therapeutic approaches [12]. miRNA17-92a-1 is a multi-functional miRNA cluster, and its dysregulation has been implicated in a wide range of human diseases, including neoplastic, cardiovascular, and immune-mediated diseases [27]. Owing to growing research regarding MIR17HG in autoimmune diseases, being the first one, we conducted this work to explore the association between the MIR17HG variant and expression and alopecia areata.

The current study showed that the AA genotype of MIR17HG variant rs4284505 conferred protection from alopecia areata, while the AG genotype was associated with disease risk and severity, including the worst DLQI score and lack of therapeutic response. Consistent with our findings, Wu et al. reported that the AA genotype of the rs4284505 variant was correlated with a better prognosis of multiple myeloma (MM) than the AG genotype associated with MM risk and worse prognosis in terms of staging and survival rate. This finding was not evidenced only at the allele level, but also at the haplotype level with other MIR17HG variants (rs1428 and rs7336610) [28]. Additionally, the A allele of such a variant was reported to have a protective effect against the susceptibility of breast cancer development in the Australian population [29]. On the other hand, on exploring the association of MIR17HG rs4284505 and systemic lupus erythematosus (SLE), Abdel-Gawad et al. found that the AG genotype conferred a protective effect against SLE development, and the GG genotype was associated with disease susceptibility and progression [17].

On the contrary, no association has been identified between the MIR17HG rs4284504 variant under additive, recessive, and dominant models and persistent apical periodontitis development or susceptibility [30]. This discrepancy in the study findings could be attributed to the ethnicity, environmental, and geographical factors, and difference in the sample size and/or methodology of previous studies. Besides, it has been noted that genetic variants could exert the underlying effects in “a cell-type-specific and context-dependent manner” [31,32].

It is worth noting that the rs4284505 variant overlaps intron one of three transcripts and exon 1 of a fourth MIR17HG splice variant. The substitution of the “A” allele by the “G” allele could disrupt the regulatory region upstream of the MIR17HG cluster, influencing the binding of multiple transcription factors with it, which, in turn, may change the gene expression level (Table S4). In addition, the rs4284505 variant was detected to be strongly in linkage disequilibrium with rs7336610 and rs1428, being significantly associated with various diseases, which might also alter the miR-17-92 cluster expression [28,33].

Using miRNA-17 as a surrogate marker of MIR17HG expression, the current study analyzed the serum level of miRNA-17 in alopecic patients compared to controls with high diagnostic performance, potentiating its role as a valuable diagnostic biomarker in alopecia areata. Regarding the correlation between the genotype and expression level, miRNA-17 was upregulated in the GG genotype, which is more associated with the severe disease phenotype than the AA or AG genotypes, indicating that the presence of the G allele (minor allele) could be associated with increased transcriptional activity of MIR17HG and, therefore, the progression of alopecia areata. Moreover, the miRNA-17 level was higher in alopecic patients with concomitant autoimmune diseases than their counterparts, indicating that such miRNA-17 overexpression may not only contribute to alopecia areata development, but could also promote the progress of multiple autoimmune disorders.

Regarding the hair disorders, miRNA17-92a-1 cluster members might contribute to female pattern hair loss (FPHL), as evidenced by the upregulation of the miRNA-92 member in the scalp skin, which was associated with abnormal fatty biosynthesis acid and metabolism as the underlying signaling pathway [34]. Wang et al. detected mmu-miR-92a upregulation in the lesional skin of an AA animal model [11]. Besides, miR-106a and miR-106b, known as members of the miRNA-17 family and paralogs of the studied cluster, have been proposed to play a crucial role in the pathogenesis of alopecia androgenetic or male pattern baldness (MPB), as their expression was markedly elevated in balding dermal papillae, being associated with the downregulation of target genes of hair follicle morphogenesis and maintenance [35,36].

The miRNA-17-92a-1 cluster and its six mature miRNAs, including miRNA-17, were markedly overexpressed in a psoriatic skin lesion, mainly in the epidermal region, compared to normal skin, and the expression level was positively correlated with the psoriasis severity index [37]. Additionally, the miRNA-17 level was upregulated explicitly in the psoriatic lesion of the imiquimod-induced model [38]. miRNA-17 was also upregulated in CD4+ T cells of patients with SLE and positively correlated with the SLE disease activity index [39], and this was evidenced in splenic T cells in a murine model of lupus [40]. In the same context, Lindberg et al. elucidated that miRNA-17 was strongly upregulated in CD4+ T cells from the peripheral blood of multiple sclerosis (MS) patients (relapsing-remitting MS) and correlated with alterations in the expression of potential target genes potentiating its contribution to MS pathogenesis [41]. miRNA-17 was also overexpressed in the tissue and serum of several hematological and solid neoplasms, and its aberrant expression level was not only correlated with tumor progression and poor prognosis, but also mediated resistance to anti-cancer treatment [42,43,44,45,46,47,48,49]. Overall, it could be argued that miRNA-17 represents a promising diagnostic biomarker and therapeutic target in various neoplastic and immune disorders.

On the contrary, a recent study reported the downregulation of miRNA-17, miRNA-20, and miRNA-106a in the serum of Egyptian SLE patients correlated with disease activity [50]. Similar findings were also detected in Swedish and Danish cohorts [51]. Cox et al. demonstrated that miRNA-17 was downregulated in whole-blood MS across all types (primary progressive, secondary progressive, and relapsing-remitting) [52]. In rheumatoid arthritis (RA), miRNA-17 under-expression was observed in the serum and synovial tissues of patients, being inversely correlated with the pathogenesis of RA [53,54]. These discrepancies in the findings could be attributed to (1) differences in the ethnic groups, disease phenotype, and study methodology, (2) variation in the cell types used for assessing miRNA-17 expression, and (3) circulatory mi-RNA being found in vesicles and or in miRNA–protein complexes, and the different centrifugation and isolation of vesicles or mi-RNA complexes could affect the assay result.

The mi-RNA 17-92a-1 cluster is a crucial modulator of the immunological and inflammatory processes. mi-RNA17-92 overexpression in lymphocytes developed autoimmunity and lymphoproliferative disease in transgenic mice through targeting BIM and PTEN genes, which assume a pivotal role in the immune tolerant mechanism in BT lymphocytes [55]. Additionally, this cluster upregulation in the DN1 stage was associated with enhanced proliferation of CD4+ cells mediating autoimmunity [27]. miRNA-17 is mainly linked to the differentiation and activation of Th1, an essential cell in autoimmune disease, promoting the production of interferon-γ [56] that signals through the JAK/STAT pathway; its inhibition is an effective therapy for various autoimmune disorders, and the JAK inhibitor has been used to reverse AA in humans and mice [11]. Furthermore, it is principally involved in enhancing Th17 cell differentiation and responses via inhibiting Ikaros family zinc finger 4 (IKZF4) [57].

On the other hand, miR-17 overexpression suppresses the differentiation of iTreg cells. It mitigates Treg cells’ immunosuppressive function through targeting the transcription factor forkhead box p3 (Foxp3) co-regulators, as well as cAMP-responsive element-binding protein1 (CREB1) and transforming growth factor B receptor 2 (TGFBR2), promoting autoimmunity [56,58]. On a related note, the imbalance of Th17 and Treg has been suggested to facilitate AA occurrence and progression [59].

Moreover, miRNA17-92a-1 is involved in B cell development (pro-B to pre-B cell transition) and differentiation, including germinal center B cells. It promotes antibody production through activating the differentiation and migration of Tfh cells to B cell follicles and germinal centers [60]. miRNA-17 promotes the macrophage phagocytic function and cytokine secretion through downregulating signal-regulatory protein α (SIRPα) [61]. Similar to other related immune-mediated skin disorders, such as psoriasis, the miRNA17-92a-1 cluster promotes chemokines, such as CXCL9 and CXCL10, in keratinocytes and further T cell chemotaxis by inhibiting the expression of suppressor of cytokine signaling 1 (SOCS1) [37].

Collectively, all of the above evidence demonstrates the pivotal role of MIR17HG in autoimmunity, which, in turn, potentiates the possible contribution of such a cluster to AA development and severity.

Although the current study was the first to address the relationship of MIR17HG SNP and gene expression with alopecia areata, some limitations could not be ignored: first, few female patients were included as they represented the sample attending the Dermatological Clinic during the study. Second, both the patients and controls were collected from outpatient clinics, so selection bias could not be avoided. Third, the expression levels were tested in patients receiving treatment with variable degrees of response. Fourth, tissue verification and the relative signaling pathway should be determined, but insufficient funding interfered with this. Hence, replication studies including both sexes with variable ethnicities will confirm the association of the specified variant with different clinical phenotypes and sex, and will delineate the precise mechanism of MIR17HG in alopecia areata.

## 5. Conclusions

A significant association of MIR17HG expression and the rs4284505A>G variant with alopecia areata was identified. This could support the role of MIR17HG in alopecia areata pathogenesis and phenotype in the Egyptian population. Larger multi-center studies in other populations are recommended to validate the findings.

## Figures and Tables

**Table 1:** Demographic characteristics of the study population.

Variables	Categories	Controls	AA	*p*-Value	OR (95% CI)
Age, years	≤30 years	144 (59)	125 (50.4)	0.05	Reference
	>30 years	100 (41)	123 (49.6)		1.41 (0.99–2.02)
Sex	Male	198 (81.1)	216 (87.1)	0.08	Reference
	Female	46 (18.9)	32 (12.9)		0.63 (0.39–1.04)
Obesity	Negative	234 (95.9)	229 (92.3)	0.12	Reference
	Positive	10 (4.1)	19 (7.7)		1.94 (0.88–4.26)
Residence	Port-Said	18 (7.4)	13 (5.2)	0.29	Reference
	Suez	22 (9)	15 (6)		0.94 (0.35–2.48)
	Ismailia	62 (25.4)	77 (31)		1.71 (0.78–3.78)
	Cairo	142 (58.2)	143 (57.7)		1.39 (0.65–2.95)
Occupation	Student	96 (39.3)	107 (43.1)	0.16	Reference
	Unemployed	100 (41)	108 (43.5)		0.96 (0.65–1.42)
	Employed	48 (19.7)	33 (13.3)		0.61 (0.36–1.03)
Family history	Alopecia	0 (0)	108 (43.5)	N.A.	
	Autoimmune disorders	0 (0)	105 (42.3)	NA.	

Data are shown as the number (percentage) or mean ± SD. A Chi-square test was used for qualitative variables, and a Student’s *t*-test was used for quantitative variables. *p*-values < 0.05 were considered statistically significant. OR (95% CI), odds ratio and confidence interval, respectively.

**Table 2:** Comparison between alopecia patients according to disease phenotype.

Characteristics		Alopecia Areata (Patchy/Aphiasis)	Alopecia Totalis/Universalis	*p*-Value	OR (95% CI)
Number		173 (69.8)	75 (30.2)		
Demographic characteristics					
Age, years	Mean ± SD	30.9 ± 7.1	30.1 ± 7.0	0.52	
Sex	Male	150 (86.7)	66 (88)	0.84	Reference
	Female	23 (13.3)	9 (12)		0.88 (0.39–2.02)
BMI, kg/m^2^	Mean ± SD	26.0 ± 2.8	25.1 ± 2.7	0.06	
Family history	Alopecia	75 (43.4)	33 (44)	0.94	1.02 (0.59–1.77)
	Autoimmune	74 (42.8)	31 (41.3)	0.88	0.94 (0.54–1.63)
Prior episode of alopecia	No	57 (32.9)	26 (34.7)	0.88	Reference
	Yes	116 (67.1)	49 (65.3)		0.92 (0.52–1.64)
Duration of disease, months	Mean ± SD	4.8 ± 9.7	4.5 ± 8.3	0.91	
Age at onset	Mean ± SD	30.5 ± 7.0	29.7 ± 7.1	0.64	
Disease characteristics					
Nail changes	Absent	125 (72.3)	38 (50.7)	**0.001**	Reference
	Present	48 (27.7)	37 (49.3)		2.53 (1.44–4.44)
Itching	Absent	137 (79.2)	52 (69.3)	0.10	Reference
	Present	36 (20.8)	23 (30.7)		1.68 (0.91–3.10)
Scalp infection	Absent	134 (77.5)	48 (64)	**0.041**	Reference
	Present	39 (22.5)	27 (36)		1.93 (1.07–3.49)
Atopy	Absent	157 (75.1)	32 (82.1)	0.41	Reference
	Present	52 (24.9)	7 (17.9)		0.66 (0.27–1.58)
Hypertension	Absent	190 (90.9)	37 (94.9)	0.54	Reference
	Present	19 (9.1)	2 (5.1)		0.54 (0.12–2.42)
Emotional stress	Absent	52 (24.9)	8 (20.5)	0.68	Reference
	Present	157 (75.1)	31 (79.5)		1.28 (0.55–2.96)
Concomitant autoimmune disease	Absent	143 (68.4)	30 (76.9)	0.34	Reference
	Present	66 (31.6)	9 (23.1)		0.65 (0.29–1.44)
SALT score	Mean ± SD	9.4 ± 8.8	13.8 ± 10.0	**<0.001**	
Prognostic score	Mean ± SD	1.61 ± 1.1	2.30 ± 1.02	**<0.001**	
DLQI score	Mean ± SD	9.4 ± 8.8	13.8 ± 10.0	**0.003**	
Responded to treatment	No	51 (29.5)	14 (18.7)	0.08	Reference
	Yes	122 (70.5)	61 (81.3)		1.82 (0.93–3.54)

Data are shown as the number (percentage) or mean ± SD. Chi-square and Fisher’s Exact tests were used for categorical variables, and Student’s *t*- and Mann–Whitney *U* tests were applied for quantitative variables. OR (95% CI), odds ratio and confidence interval between alopecia totalis and universalis versus patchy and aphiasis alopecia areata, respectively. BMI: body mass index; SALT: Severity of Alopecia Tool score for severity assessment; and DLQI: Dermatology Life Quality Index questionnaire. Bold values indicate statistically significant *p*-values of <0.05.

**Table 3:** Genotype and allele frequencies of MIR17 rs4284505 polymorphism.

	All Subjects		Controls		Cases		
**Variable**	Count	Proportion	Count	Proportion	Count	Proportion	*p*-value
**Allele**							
G	575	0.58	291	0.60	284	0.57	0.45
A	409	0.42	197	0.40	212	0.43	
**Genotypes**							
A/A	84	0.17	46	0.19	38	0.15	**0.032**
G/A	241	0.49	105	0.43	136	0.55	
G/G	167	0.34	93	0.38	74	0.30	
**HWE**							
*p*-value	0.93		0.11		0.07		

Values are shown as numbers (%). HWE: Hardy–Weinberg Equilibrium. A Chi-square test was used. Bold values indicate statistically significant *p*-values of <0.05. Comparison between G/A versus A/A: *p* = 0.07, G/G versus A/A: *p* = 0.88, and G/G versus G/A: *p* = 0.016.

**Table 4:** Risk of alopecia areata by genetic association models of MIR17 rs4284505 genotypes.

Model	Genotype	Controls	Cases	Crude OR (95% CI)	*p*-Value	Adjusted OR (95% CI)	*p*-Value
Codominant	G/G	93 (38.1%)	74 (29.8%)	1.00	**0.032**	1.00	**0.044**
	A/G	105 (43%)	136 (54.8%)	1.63 (1.09–2.42)		1.36 (0.84–2.20)	
	A/A	46 (18.9%)	38 (15.3%)	1.04 (0.61–1.76)		0.60 (0.30–1.20)	
Dominant	G/G	93 (38.1%)	74 (29.8%)	1.00	0.052	1.00	0.66
	A/G–A/A	151 (61.9%)	174 (70.2%)	1.45 (1.00–2.11)		1.11 (0.70–1.74)	
Recessive	G/G–A/G	198 (81.2%)	210 (84.7%)	1.00	0.30	1.00	**0.03**
	A/A	46 (18.9%)	38 (15.3%)	0.78 (0.49–1.25)		0.51 (0.27–0.96)	
Overdominant	G/G–A/A	139 (57%)	112 (45.2%)	1.00	**0.008**	1.00	**0.043**
	A/G	105 (43%)	136 (54.8%)	1.61 (1.13–2.29)		1.57 (1.01–2.45)	
Log-additive	---	---	---	1.10 (0.86–1.42)	0.45	0.88 (0.64–1.20)	0.41

Values are shown as numbers (%). A Chi-square test was used. OR (95% CI), odds ratio and confidence interval, respectively. Bold values indicate statistically significant *p*-values of <0.05. Adjusted covariates: age, gender, BMI, and occupation.

## Data Availability

All generated data in this study are included in the article.

## Supplementary Materials

The following supporting information can be downloaded at: https://www.mdpi.com/article/10.3390/genes13030505/s1, Table S1: MicroRNA 17-92a cluster is involved in hair follicle-related gene ontology (GO); Table S2: Functional enrichment analysis of the microRNAs 17-92a cluster; Table S3: Association of miR-17 rs4284505 genotypes and miR-17 expression with clinical features; Table S4: Functional impact of MIR17HG polymorphism on regulatory regions. Figure S1. (A) Allele discrimination plot (single-nucleotide polymorphism assay, rs4284505) using TaqMan genotyping polymerase chain reaction (StepOne Real-Time; Applied Biosystems, Thermo Fisher Scientific, Foster City, CA, USA). Alleles, such as homozygous A/A (lower right red cluster), heterozygous A/G (middle green cluster), and homozygous G/G (upper left blue cluster), were retrieved using allele-calling software. The negative control samples were represented as black squares (x: undetermined) in the lower left corner of the plot. (B) Amplification plot for the quantification of MIR17HG by the Real-Time TaqMan^®^ PCR assay showing the of change in gene expression plotted vs. the PCR cycle number. This plot was obtained from the AB 7500HT instrument (Applied Biosystems, USA).
