# Supplementary material for: MicroRNA-17-92a-1 Host Gene (MIR17HG) Expression Signature and rs4284505 Variant Association with Alopecia Areata: A Case–Control Study
Source: Genes (Basel). 2022 Mar 12;13(3):505. doi: 10.3390/genes13030505 (PMC8955921; doi:10.3390/genes13030505)
Supplement: Supplementary file 1 [file genes-13-00505-s001.zip › genes-1615121-supplementary.pdf]

Supplementary Table S1. MicroRNA 17-92a cluster is involved in hair follicle related gene ontology (GO) terms

Go term	ID	Rank	microRNA	# genes	Target genes	Method	P value
Hair cycle process	GO: 00224405	6	miR-20a-3p	2	**SOX18**	HITS-CLIP	1.21E-07
					**CTNNB1**	HITS-CLIP	
		9	miR-17-3P	2	**ERCC2**	HITS-CLIP	1.21E-07
					**CTNNB1**	Multiple	
		132	miR-92a-3p	6	**DICER1**	Multiple	5.24E-04
					**CELSR1**		
					**FZD6**	HITS-CLIP	
					**TP63**		
					**VANGL2**		
					**AARS**	Multiple	
Hair follicle development	GO: 0001942	43	miR-92a-3p	6	**DICER1**	Multiple	2.86E-11
					**CELSR1**		
					**FZD6**	HITS-CLIP	
					**TP63**		
					**VANGL2**		
					**AARS**	Multiple	
		60	miR-19a-3p	6	**DICER1**	Multiple	2.86E-11
					**CELSR1**	HITS-CLIP	
					**FZD6**	HITS-CLIP	
					**INHBA**	PAR-CLIP	
					**EDARADD**	Multiple	
					**DKK1**	HITS-CLIP	
		127	miR-19b-3p	4	**DICER1**	Multiple	3.25E-07
					**CELSR1**	HITS-CLIP	
					**INHBA**	PAR-CLIP	
					**EDARADD**	Multiple	
		117	miR-20a-5p	4	**DICER1**	HITS-CLIP	3.25E-07
					**ACVR1B**	Multiple	
					**FZD6**	PAR-CLIP	
					**CD109**	HITS-CLIP	
		154	miR-18a-3p	3	**DICER1**	Multiple	2.49E-05
					**FZD3**	PAR-CLIP	
					**EDARADD**		
		174	miR-17-5p	3	**DICER1**	Multiple	2.49E-05
					**FZD6**	Multiple	
					**TFAP2C**	Multiple	
Hair follicle morphogenesis	GO: 0031069	11	miR-17-3p	6	**IGFBP5**	HITS-CLIP	2.98E-13
					**DICER1**	Multiple	
					**BCL2**	PAR-CLIP	
					**NOTCH1**	HITS-CLIP	
					**CTNNB1**	Multiple	
					**CDC42**	HITS-CLIP	
		48	miR-17-5p	5	**IGFBP5**	PAR-CLIP	
					**DICER1**	Multiple	
					**RUNX1**	Multiple	
					**BCL2**	Multiple	
					**RUNX3**	Multiple	

		63	miR-92a-3p	4	**IGFBP5**	HITS-CLIP	1.60E-08
					**DICER1**	Multiple	
					**TP63**		
					**CTNNB1**	Multiple	
		202	miR-18a	2	**DICER1**	Multiple	3.19E-04
					**RUNX1**	Multiple	
		269	miR-19a-3p	2	**DICER1**	Multiple	3.19E-04
					**FGF7**	HITS-CLIP	
		372	miR-18a-3p	1	**DICER1**	Multiple	2.54E-02
		506	miR-92a-1-5p	1	**DICER1**	PAR-CLIP	2.54E-02
Hair follicle cell proliferation	GO: 0071335	54	miR-92a-1-5p	1	**DICER1**	Multiple	3.28E-05
		95	miR-19b-3p	1	**DICER1**	Multiple	3.28E-05
		98	miR-18a-3p	1	**DICER1**	Multiple	3.28E-05
		120	miR-19a-3p	1	**DICER1**	Multiple	3.28E-05
		148	miR-17-5p	1	**DICER1**	Multiple	3.28E-05
		206	miR-17-3p	1	**DICER1**	Multiple	3.28E-05
		208	miR-20a-5p	1	**DICER1**	Multiple	3.28E-05
Hair follicle placode formatio	GO: 0060789	1	miR-17-3p	5	**GNAS**	Multiple	2.99E-18
					**HDAC1**	PAR-CLIP	
					**CTNNB1**	Multiple	
					**HDAC2**	HITS-CLIP	
					**CDC42**	HITS-CLIP	
		6	miR-92a-3p	3	**HDAC1**	PAR-CLIP	1.22E-10
					**CTNNB1**	Multiple	
					**HDAC2**	PAR-CLIP	
		139	miR-18a-3p	1			9.91E-04
		225	miR-17-5p	1			9.91E-04
		228	20a-3p	1	**CTNNB1**	HITS-CLIP	9.91E-04
Hair follicle maturation	GO: 0048820	1	miR-17-5p	2	**ERCC2**	HITS-CLIP	1.89E-09
					**RBPJ**	Multiple	
		14	miR-92a-3p	1	**RBPJ**	Multiple	7.37E-05
		31	miR-92a-1-5p	1	**RBPJ**	Multiple	7.37E-05
		33	miR-18a-3p	1	**RBPJ**	HITS-CLIP	7.37E-05
		68	miR-18a-5p	1	**RBPJ**	HITS-CLIP	7.37E-05
		117	miR-19b-3p	1	**RBPJ**	HITS-CLIP	7.37E-05
		126	miR-17-3p	1	**ERCC2**	HITS-CLIP	7.37E-05
		128	miR-20a-5p	1	**RBPJ**	HITS-CLIP	7.37E-05
Hair cell diffrentiation	GO: 0035315	13	miR-17-3p	2	**ERCC2**	HITS-CLIP	1.55E-07
					**CTNNB1**	Multiple	
		23	miR-19b-1-5p	1	**ERCC3**	Multiple	5.92E-04
		36	miR-92a-3p	1	**ERCC3**	Multiple	5.92E-04
		100	miR-17-5p	1	**ERCC2**	Multiple	5.92E-04
		122	miR-19a-5p	1	**ERCC3**	PAR-CLIP	5.92E-04
Hair cycle	GO: 0042633	25	miR-92a-3p	1	**SNRPE**	HITS-CLIP	2.23E-03
		87	miR-17-5p	1	**PPP1R13L**	HITS-CLIP	2.23E-03

Supplementary Table S2. Functional enrichment analysis of the microRNAs 17-92a cluster

KEGG pathway	p-value	#genes	#miRNAs
**Proteoglycans in cancer**	**1.0e-325**	97	6
**TGF-beta signaling pathway**	**4.55E-15**	43	7
**Chronic myeloid leukemia**	**7.54E-13**	46	6
**Hepatitis B**	**7.83E-13**	68	4
**Glioma**	**1.22E-11**	32	4
**Hippo signaling pathway**	**1.95E-11**	72	7
**Adherens junction**	**4.48E-11**	38	5
**Viral carcinogenesis**	**1.08E-10**	75	5
**Pathways in cancer**	**1.09E-10**	139	4
**Cell cycle**	**7.91E-10**	52	6
**FoxO signaling pathway**	**3.96E-09**	63	4
**Thyroid hormone signaling pathway**	**6.66E-09**	63	4
**Bladder cancer**	**1.37E-08**	27	6
**p53 signaling pathway**	**1.79E-08**	37	5
**Bacterial invasion of epithelial cells**	**2.03E-08**	39	4
**ECM-receptor interaction**	**2.26E-08**	18	2
**Fatty acid biosynthesis**	**2.95E-08**	1	1
**Prion diseases**	**3.80E-08**	6	1
**Colorectal cancer**	**5.34E-07**	35	4
**Prostate cancer**	**1.42E-06**	41	3
**Focal adhesion**	**2.19E-06**	90	5
**Regulation of actin cytoskeleton**	**3.13E-06**	86	5
**Prolactin signaling pathway**	**3.95E-06**	34	3
**Pancreatic cancer**	**3.32E-05**	31	3
**Lysine degradation**	**4.76E-05**	18	5
**Signaling pathways regulating pluripotency of sten**	**4.99E-05**	62	4
**Protein processing in endoplasmic reticulum**	**5.92E-05**	55	2
**Sphingolipid signaling pathway**	**7.53E-05**	49	3
**RNA transport**	**0.000102**	58	3
**Melanoma**	**0.000134**	25	3
**RNA degradation**	**0.000135**	30	5
**mTOR signaling pathway**	**0.000213**	28	3
**Transcriptional misregulation in cancer**	**0.000319**	55	3
**mRNA surveillance pathway**	**0.00034**	36	2
**Sphingolipid metabolism**	**0.000607**	8	2
**Non-small cell lung cancer**	**0.000611**	26	3
**Fatty acid metabolism**	**0.002094**	9	3
**Insulin signaling pathway**	**0.002313**	52	3
**Renal cell carcinoma**	**0.002908**	26	2
**Estrogen signaling pathway**	**0.004091**	40	3
**Ubiquitin mediated proteolysis**	**0.004364**	46	3
**Endometrial cancer**	**0.005013**	24	2
**Circadian rhythm**	**0.005096**	15	3
**Thyroid cancer**	**0.006483**	16	4
**Small cell lung cancer**	**0.007543**	26	2
**Shigellosis**	**0.008276**	19	1

**Fatty acid elongation**	**0.008908**	2	1
**Central carbon metabolism in cancer**	**0.012451**	23	2
**Axon guidance**	**0.01594**	42	3
**Oocyte meiosis**	**0.016976**	32	2
**Adrenergic signaling in cardiomyocytes**	**0.017312**	44	3
**Neurotrophin signaling pathway**	**0.020944**	38	2
**Long-term depression**	**0.024704**	19	3
**Endocytosis**	**0.031641**	62	3
**Acute myeloid leukemia**	**0.042271**	23	3
**PI3K-Akt signaling pathway**	**0.046219**	96	3

GO Category	p-value	#genes	#miRNAs
**transforming growth factor beta receptor signaling pathway**	**0**	80	4
**cellular component disassembly involved in execution phase of apo**	**0**	30	5
**platelet activation**	**0**	70	5
**innate immune response**	**0**	199	5
**phosphatidylinositol-mediated signaling**	**0**	46	5
**positive regulation of protein insertion into mitochondrial membrar**	**0**	21	5
**G2/M transition of mitotic cell cycle**	**0**	65	6
**Fc-gamma receptor signaling pathway involved in phagocytosis**	**0**	41	6
**immune system process**	**0**	436	7
**transcription initiation from RNA polymerase II promoter**	**0**	92	7
**fibroblast growth factor receptor signaling pathway**	**0**	76	7
**protein complex assembly**	**0**	259	8
**blood coagulation**	**0**	174	8
**intrinsic apoptotic signaling pathway**	**0**	36	8
**DNA metabolic process**	**0**	282	9
**transcription, DNA-templated**	**0**	750	9
**epidermal growth factor receptor signaling pathway**	**0**	85	9
**cellular component assembly**	**0**	445	9
**mitotic cell cycle**	**0**	206	10
**cellular protein modification process**	**0**	925	10
**response to stress**	**0**	768	10
**biological_process**	**0**	4652	10
**cell death**	**0**	366	10
**catabolic process**	**0**	742	10
**biosynthetic process**	**0**	1441	10
**gene expression**	**0**	343	10
**viral process**	**0**	268	10
**RNA metabolic process**	**0**	122	10
**mRNA metabolic process**	**0**	114	10
**cellular nitrogen compound metabolic process**	**0**	1784	10
**nucleobase-containing compound catabolic process**	**0**	371	10
**Fc-epsilon receptor signaling pathway**	**0**	80	10
**cellular protein metabolic process**	**0**	214	10
**small molecule metabolic process**	**0**	799	10
**symbiosis, encompassing mutualism through parasitism**	**0**	296	10
**neurotrophin TRK receptor signaling pathway**	**0**	134	10
**membrane organization**	**0**	261	10
**macromolecular complex assembly**	**0**	330	10
**cellular lipid metabolic process**	**1.11E-16**	65	8
**cell cycle**	**7.77E-16**	282	6
**toll-like receptor 10 signaling pathway**	**6.77E-15**	31	6
**mRNA processing**	**1.41E-14**	183	9
**viral life cycle**	**1.74E-13**	50	8
**nucleocytoplasmic transport**	**3.92E-13**	113	5
**toll-like receptor TLR1:TLR2 signaling pathway**	**6.69E-13**	30	6
**toll-like receptor TLR6:TLR2 signaling pathway**	**6.69E-13**	30	6

G1/S transition of mitotic cell cycle	9.76E-13	79	7
insulin receptor signaling pathway	1.16E-12	59	4
chromatin organization	2.01E-12	56	6
toll-like receptor 5 signaling pathway	2.73E-12	31	6
TRIF-dependent toll-like receptor signaling pathway	4.73E-12	34	6
activation of signaling protein activity involved in unfolded protein r	8.98E-12	32	5
toll-like receptor 9 signaling pathway	1.36E-11	32	6
nuclear-transcribed mRNA catabolic process, deadenylation-depend	1.66E-11	27	6
DNA damage response, signal transduction by p53 class mediator re	1.79E-11	38	6
apoptotic signaling pathway	1.93E-11	53	5
mitotic nuclear envelope disassembly	3.15E-11	20	5
platelet degranulation	8.75E-11	25	4
termination of RNA polymerase II transcription	3.09E-10	25	6
regulation of transcription from RNA polymerase II promoter in resp	3.24E-10	15	4
MyD88-independent toll-like receptor signaling pathway	5.36E-10	30	5
stress-activated MAPK cascade	6.26E-10	26	4
cytoskeleton organization	1.17E-09	155	4
cell junction organization	1.29E-09	48	3
ribonucleoprotein complex assembly	2.88E-09	43	4
in utero embryonic development	5.49E-09	108	4
RNA splicing	1.31E-08	101	6
toll-like receptor 3 signaling pathway	2.98E-08	28	4
toll-like receptor 2 signaling pathway	3.54E-08	26	4
toll-like receptor 4 signaling pathway	3.55E-08	38	6
apoptotic process	3.77E-08	195	3
toll-like receptor signaling pathway	4.15E-08	35	4
vesicle-mediated transport	6.81E-08	210	3
cellular component movement	7.09E-08	36	4
negative regulation of transcription from RNA polymerase II promot	1.11E-07	232	4
cell cycle arrest	1.18E-07	43	2
NLS-bearing protein import into nucleus	2.32E-07	11	7
transcription from RNA polymerase II promoter	4.43E-07	147	4
regulation of ubiquitin-protein ligase activity involved in mitotic cell	4.56E-07	31	5
protein polyubiquitination	4.81E-07	55	4
intracellular transport of virus	5.05E-07	11	5
mRNA 3'-end processing	6.36E-07	20	3
post-translational protein modification	1.03E-06	55	4
positive regulation of ubiquitin-protein ligase activity involved in mi	2.02E-06	28	4
hexose transport	3.98E-06	22	6
generation of precursor metabolites and energy	7.36E-06	89	5
protein targeting	1.06E-05	70	4
regulation of glucose transport	1.14E-05	17	5
mRNA splicing, via spliceosome	1.19E-05	65	4
negative regulation of transcription, DNA-templated	1.29E-05	168	3
positive regulation of apoptotic process	1.39E-05	118	3
protein ubiquitination	2.27E-05	123	3
antigen processing and presentation of exogenous peptide antigen v	4.05E-05	37	4
cell proliferation	7.3E-05	135	3
JAK-STAT cascade involved in growth hormone signaling pathway	7.36E-05	13	3
nuclear-transcribed mRNA poly(A) tail shortening	8.5E-05	15	4

**androgen receptor signaling pathway**	**8.68E-05**	25	4
**endoplasmic reticulum unfolded protein response**	**9.74E-05**	32	3
**protein N-linked glycosylation via asparagine**	**0.000111**	28	3
**axon guidance**	**0.000131**	106	3
**positive regulation of transcription, DNA-templated**	**0.000179**	214	4
**MyD88-dependent toll-like receptor signaling pathway**	**0.000203**	26	3
**nucleotide-binding domain, leucine rich repeat containing receptor s**	**0.000395**	14	3
**cell motility**	**0.000411**	73	2
**cell junction assembly**	**0.000486**	20	2
**regulation of defense response to virus by virus**	**0.00053**	13	3
**negative regulation of cell proliferation**	**0.000695**	112	2
**regulation of cell cycle**	**0.000855**	52	3
**regulation of nitric-oxide synthase activity**	**0.001055**	5	2
**negative regulation of transforming growth factor beta receptor sign**	**0.00129**	35	3
**positive regulation of nuclear-transcribed mRNA catabolic process, c**	**0.001311**	8	2
**anaphase-promoting complex-dependent proteasomal ubiquitin-de**	**0.001314**	22	2
**regulation of cellular amino acid metabolic process**	**0.001463**	16	2
**Notch signaling pathway**	**0.001474**	48	3
**signal transduction**	**0.001648**	774	2
**extracellular matrix organization**	**0.001679**	53	2
**post-Golgi vesicle-mediated transport**	**0.001787**	10	2
**positive regulation of nuclear-transcribed mRNA poly(A) tail shorter**	**0.0018**	8	4
**nitric oxide metabolic process**	**0.002048**	4	2
**extracellular matrix disassembly**	**0.002346**	29	3
**activation of MAPKK activity**	**0.00252**	27	3
**glycerophospholipid biosynthetic process**	**0.002782**	19	3
**negative regulation of ubiquitin-protein ligase activity involved in m**	**0.0029**	14	2
**COPI coating of Golgi vesicle**	**0.003121**	6	2
**nucleobase-containing small molecule metabolic process**	**0.003443**	21	2
**cellular response to hypoxia**	**0.003892**	35	3
**regulation of transcription involved in G1/S transition of mitotic cell**	**0.004854**	9	2
**leukocyte migration**	**0.006869**	25	2
**nuclear-transcribed mRNA catabolic process, nonsense-mediated de**	**0.010482**	19	1
**inositol phosphate metabolic process**	**0.012437**	13	3
**SRP-dependent cotranslational protein targeting to membrane**	**0.01485**	11	2
**viral transcription**	**0.016991**	8	2
**negative regulation of translation**	**0.01851**	18	2
**protein maturation**	**0.020399**	32	2
**anatomical structure morphogenesis**	**0.021381**	29	2
**stem cell maintenance**	**0.023488**	13	1
**positive regulation of viral transcription**	**0.024556**	14	2
**negative regulation of apoptotic process**	**0.025377**	73	1
**positive regulation of type I interferon production**	**0.026091**	9	1
**regulation of small GTPase mediated signal transduction**	**0.033218**	54	3
**virion assembly**	**0.036658**	11	2
**protein import into nucleus**	**0.048113**	13	1
**viral protein processing**	**0.049742**	8	2

Supplementary Table S3. Association of MIR17 rs4284505 genotypes and MIR17 expression with clinical features

Characteristics		AA	AG	GG	*p*- value	MIR-17 expression	*p*- value
Number		38	136	74			
Demographic characteristics							
Age, year	≤30 years	17 (44.7)	72 (52.9)	36 (48.6)	**<0.001**	17.7 (1.16-343)	0.169
	>30 years	21 (55.3)	64 (47.1)	38 (51.4)		58.3 (5.34-1068)	
Sex	Male	35 (92.1)	118 (86.8)	63 (85.1)	0.69	35.8 (2.02-470.4)	0.357
	Female	3 (7.9)	18 (13.2)	11 (14.9)		6.15 (0.05-1906)	
Obesity	Absent	33 (86.8)	127 (93.4)	69 (93.2)	0.44	28.2 (1.68-476.3)	0.60
	Present	5 (13.2)	9 (6.6)	5 (6.8)		127 (11.4-568.9)	
Residence	Port-said	1 (2.6)	7 (5.1)	5 (6.8)	0.07	476.3 (373-2704)	0.052
	Suez	2 (5.3)	10 (7.4)	3 (4.1)		85.9 (15.1-1429)	
	Ismailia	12 (31.6)	42 (30.9)	23 (31.1)		6.9 (0.56-66.2)	
	Cairo	23 (60.5)	77 (56.6)	43 (58.1)		43.5 (1.95-809)	
Occupation	Student	18 (47.4)	62 (45.6)	27 (36.5)	**0.005**	22.5 (1.57-337.6)	0.553
	Unemployed	19 (50)	56 (41.2)	33 (44.6)		27.9 (2.02-578.1)	
	Employed	1 (2.6)	18 (13.2)	14 (18.9)		66.8 (3.1-2512)	
Family history	Alopecia	21 (55.3)	63 (46.3)	24 (32.4)	**0.044**	32.4 (1.68-1321)	0.534
	Autoimmune	20 (52.6)	60 (44.1)	25 (33.8)	0.13	85.9 (1.68-1370)	0.304
Prior episode of alopecia	No	8 (21.1)	40 (29.4)	35 (47.3)	**0.007**	36 (3.2-1784)	0.364
	Yes	30 (78.9)	96 (70.6)	39 (52.7)		22.6 (1.7-452.7)	
Duration of disease	≤ 12 months	32 (84.2)	113 (83.1)	65 (87.8)	0.65	29.4 (1.7-494.8)	0.801
	>12 months	6 (15.8)	23 (16.9)	9 (12.2)		39.9 (3.5-657.7)	
Age at onset	≤ 20 years	35 (92.1)	127 (93.4)	63 (85.1)	0.13	28.2 (1.8-452.7)	0.471
	>20 years	3 (7.9)	9 (6.6)	11 (14.9)		66.3 (0.4-3239.6)	
Disease characteristics							
Alopecia subtype	Patchy	35 (92.1)	118 (86.8)	56 (75.7)	**0.038**	13 (1.6-476.3)	0.165
	Others	3 (7.9)	18 (13.2)	18 (24.3)		66.9 (18.7-2299.6)	
Nail changes	Absent	27 (71.1)	89 (65.4)	47 (63.5)	0.72	35.7 (2.1-532.8)	0.93
	Present	11 (28.9)	47 (34.6)	27 (36.5)		27.8 (1.7-501.5)	
Itching	Absent	31 (81.6)	96 (70.6)	62 (83.8)	0.07	36 (4.3-531.9)	0.070
	Present	7 (18.4)	40 (29.4)	12 (16.2)		8 (0.1-532.8)	
Scalp infection	Absent	28 (73.7)	90 (66.2)	64 (86.5)	**0.006**	36 (1.7-452.7)	0.936

	Present	10 (26.3)	46 (33.8)	10 (13.5)		10.7 (1.9-810)	
Atopy	Absent	34 (89.5)	98 (72.1)	57 (77)	0.08	36 (1.9-810)	0.522
	Present	4 (10.5)	38 (27.9)	17 (23)		22.6 (1.3-228.4)	
Hypertension	Absent	30 (78.9)	127 (93.4)	70 (94.6)	**0.010**	27.8 (1.7-452.7)	0.262
	Present	8 (21.1)	9 (6.6)	4 (5.4)		123 (1.7-2234.1)	
Emotional stress	Absent	8 (21.1)	38 (27.9)	14 (18.9)	0.30	35.8 (1.9-1224.8)	0.580
	Present	30 (78.9)	98 (72.1)	60 (81.1)		29.4 (1.7-458.6)	
Concomitant autoimmune disease	Absent	22 (57.9)	98 (72.1)	53 (71.6)	0.22	9.7 (0.6-289.8)	**0.049**
	Present	16 (42.1)	38 (27.9)	21 (28.4)		112.6 (4.1-1189.6)	
Responded to treatment	No	5 (13.2)	44 (32.4)	16 (21.6)	**0.033**	66.9 (1.7-1465.8)	0.189
	Yes	33 (86.8)	92 (67.6)	58 (78.4)		11 (1.7-452.7)	

Data are shown as number (percentage) or as medians (upper-lower limits). Chi-square and Fisher's Exact tests were used for categorical variables, and Mann-Whitney U test was applied for quantitative variables. Bold values indicate statistical significance at *p*-value < 0.05.

Supplementary Table S4. Functional impact of MIR17HG polymorphism on regulatory regions.

Consequence type	Transcription factors	Motif position	Motif score change
TF binding site	FOXO1::ELF1, FOXJ2::ELF1, FOXJ3::ELF1	13 (out of 15)	Less like consensus sequence
TF binding site	ELF1, ELF4, ELF5, ELF3, ELF2, ETV7	3 (out of 12)	More like consensus sequence
TF binding site	ETV2::DLX3, HOXB2::ELF1, HOXB2::ELK3	9 (out of 18)	Less like consensus sequence
TF binding site	E2F1::ELK1	14 (out of 22)	Less like consensus sequence
TF binding site	ETV2::PAX5, ELK1::PAX1, ELK1::PAX5, ELK1::PAX9	1 (out of 20)	Less like consensus sequence
TF binding site	HOXB2::ELF1	8 (out of 17)	Less like consensus sequence
TF binding site	ETV2::HOXA2, FLI1::DLX2, HOXB2::ELF1, HOXB2::ELK3, ETV2::DRGX, ELK1::HOXA1, FLI1::DRGX, ETV5::DRGX, ETV5::HOXA2, HOXB2::ELK1	1 (out of 14)	Less like consensus sequence
TF binding site	HOXB2::ELF1	13 (out of 22)	More like consensus sequence
TF binding site	HOXA3::PAX5	14 (out of 20)	Less like consensus sequence

Motif position is the position of the variant within the motif, in base pair coordinates, from the start of the motif.
The motif scores are between 0 and 1. It indicates how strong a binding site it is for the transcription factor, with 1 the best and 0 the worst. The score change indicates whether the score increases or decreases and by how much as a result of the SNP. It indicates whether the SNP changes a base which is vital to the motif function or not.

Data source: ensembl.org

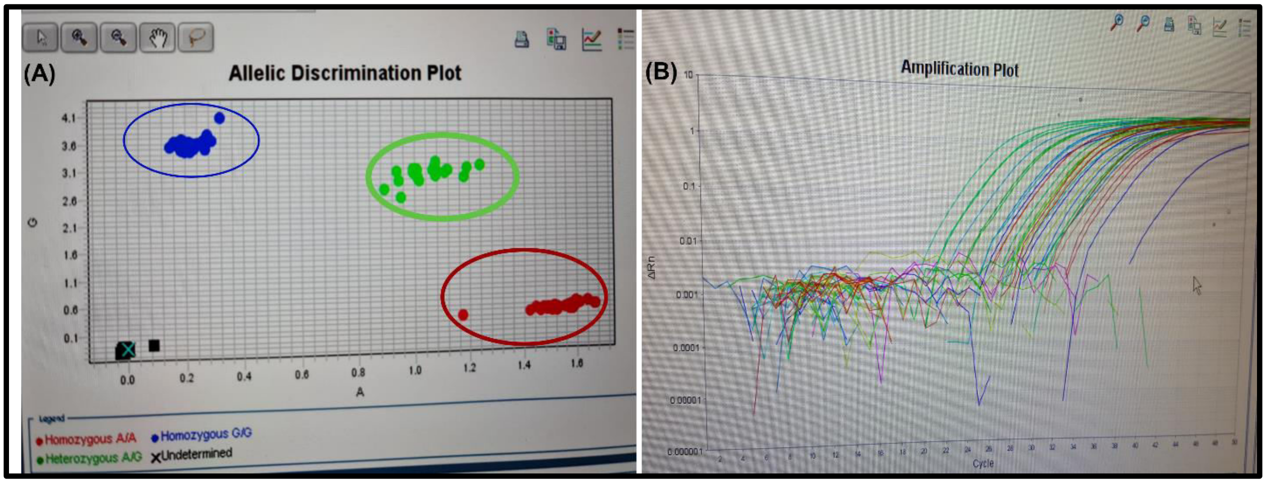

Figure S1. (A) Allele discrimination plot (single nucleotide polymorphism assay, rs4284505) using TaqMan genotyping polymerase chain reaction (StepOne Real-Time; Applied Biosystems, Thermo Fisher Scientific, Foster City, CA, USA). Alleles as homozygous A/A (lower right red cluster), heterozygous A/G (middle green cluster) and homozygous G/G (upper left blue cluster) were retrieved using allele calling software. The negative control samples were represented as black squares (x: undetermined) in the lower left corner of the plot. (B) Amplification plot for the quantification of MIR17HG by the Real-Time TaqMan® PCR assay shows log of change in gene expression plotted vs. PCR cycle number. This plot from the AB 7500HT instrument (Applied Biosystems, USA).
